# Molecular detection of cytomegalovirus in hospitalized children: a cross-sectional study in Makassar, Indonesia

**DOI:** 10.11604/pamj.2026.53.138.51023

**Published:** 2026-03-24

**Authors:** Hariati Hamzah, Fadhilah Syamsuri, Nadyah Haruna, Mochammad Hatta, Lisa Tenriesa Muslich, Yoeke Dewi Rasita, Baedah Madjid

**Affiliations:** 1 Department of Clinical Microbiology, Faculty of Medicine, Hasanuddin University, Makassar, Indonesia,; 2 Clinical Microbiology Laboratory, Hasanuddin University Hospital, Makassar, Indonesia,; 3 Microbiology Laboratory, Wahidin Sudirohusodo Hospital, Makassar, Indonesia,; 4 School of Medicine, Faculty of Medicine and Health Science, Alauddin State Islamic University, Makassar, Indonesia,; 5 Center for Health Laboratory, Makassar, South Sulawesi, Indonesia

**Keywords:** Cytomegalovirus, polymerase chain reaction, viremia, serological tests, infant

## Abstract

**Introduction:**

Human cytomegalovirus (HCMV) is a significant pathogen in pediatric populations, yet serological diagnosis lacks specificity. This study aimed to identify active CMV infection through molecular methods in hospitalized children previously diagnosed serologically.

**Methods:**

a hospital-based descriptive cross-sectional study was conducted from December 2024 to March 2025 at Wahidin Sudirohusodo Hospital, a tertiary referral and teaching hospital in Makassar, Indonesia. Blood samples from 50 pediatric inpatients with serological evidence suggesting CMV exposure were subjected to conventional polymerase chain reaction (PCR) targeting the HindIII-X fragment (406 bp). PCR-positive samples were sequenced for local phylogenetic description. Descriptive statistics assessed molecular findings stratified by demographics, serology, and clinical manifestations.

**Results:**

of 50 blood samples examined, 9 (18%) were positive for CMV DNA by PCR. Positivity rates were higher in male children (21.4%) compared to females (13.6%), with the highest prevalence in infants <1 year (24.1%). Among serology-reactive cases, the highest detection rate was observed in patients with concurrent IgM and IgG reactivity (50%), compared to IgG alone (10.3%). Phylogenetic analysis of HindIII-X sequences revealed two descriptive, well-supported clades (bootstrap 98-99%) within human betaherpesvirus 5 in this hospital cohort. Notably, 82% of serologically positive samples were negative for CMV DNA in blood, suggesting limited systemic viremia despite positive antibody status; CMV replication may still occur in end-organ compartments without being detected in peripheral blood, particularly when using conventional PCR with lower analytic sensitivity than quantitative assays.

**Conclusion:**

molecular confirmation via PCR is essential to distinguish active CMV infection from serological evidence of past or latent infection. The substantial discordance between serology and blood PCR findings supports integration of molecular testing as a confirmatory standard in pediatric CMV evaluation, particularly in IgM-positive cases-to prevent unnecessary antiviral therapy and strengthen antimicrobial stewardship in resource-limited, high-seroprevalence settings.

## Introduction

Human cytomegalovirus (HCMV), classified as human herpesvirus 5 (HHV-5), is a double-stranded DNA virus belonging to the subfamily betaherpesvirinae and exhibits strict species specificity [1]. HCMV possesses the largest genome among human herpesviruses, approximately 235 kilobases in length, encoding numerous proteins that facilitate replication across diverse cell types, including fibroblasts, epithelial cells, and endothelial cells, while enabling evasion of host immune surveillance [2]. A defining biological feature of HCMV infection is its capacity to establish lifelong latency, predominantly within CD34+ hematopoietic progenitor cells and cells of the myeloid lineage, with the potential for reactivation during periods of immune dysfunction or immunosuppression [3]. Although infection is often asymptomatic in immunocompetent individuals, HCMV represents a major cause of morbidity and mortality in vulnerable populations, particularly neonates and immunocompromised patients, including those living with HIV/AIDS [4].

Globally, HCMV seroprevalence is strongly associated with socioeconomic conditions, with substantially higher rates observed in low- and middle-income countries compared with high-income settings, contributing to an increased burden of congenital infection [5,6]. In Indonesia, the high background seroprevalence of HCMV presents significant diagnostic challenges, particularly in distinguishing latent infection from active disease due to variability in clinical presentation and diagnostic approaches [7,8]. Previous studies have demonstrated an association between congenital HCMV infection and sensorineural hearing impairment among Indonesian children [9]. However, diagnostic discordance remains common, with documented discrepancies between serological findings and molecular detection of HCMV DNA, particularly in infants, highlighting the limitations of conventional serological testing [10]. In settings with very high HCMV seroprevalence, the implementation of more precise and evidence-based diagnostic standards is therefore critically needed [9,10].

Both vertical and horizontal transmission of HCMV in children can result in viremia and lytic replication within visceral organs [11,12]. Although cellular immune responses, particularly those mediated by CD8+ T lymphocytes and natural killer cells, play a crucial role in suppressing viral replication, HCMV is not eradicated and persists in a latent state with the potential for reactivation during immune dysfunction [13]. Reactivation of HCMV has been shown to induce sustained expansion and functional differentiation of the NK-cell repertoire, reflecting ongoing host-virus immune interaction despite apparent immune control [14]. In hospitalized pediatric patients, HCMV reactivation may complicate underlying illnesses, including severe respiratory infections, thereby contributing to poorer clinical outcomes [15]. If inadequately controlled, HCMV infection can progress to serious complications such as sensorineural hearing loss, neurological impairment, hepatitis, and, in severe cases, multi-organ dysfunction [16,17].

A major clinical challenge lies in the accurate confirmation of active HCMV infection. Serological assays detecting anti-HCMV IgM and IgG antibodies, which are widely used in resource-limited settings, have well-recognized limitations [18,19]. Anti-HCMV IgM lacks optimal specificity due to its persistence following primary infection and potential cross-reactivity with other herpesviruses, including Epstein-Barr virus [20]. Importantly, antiviral therapies commonly used for HCMV infection, such as ganciclovir and valganciclovir, are associated with significant hematologic and renal toxicities, including neutropenia and thrombocytopenia [21]. Consequently, initiation of antiviral treatment based solely on serological findings, without molecular confirmation of active viral replication, may expose pediatric patients to unnecessary drug-related risks [22].

Quantitative PCR (qPCR) is considered the gold standard for CMV DNA detection and viral load monitoring because it offers higher analytic sensitivity and allows quantification of DNAemia [23]. However, routine qPCR remains limited in many tertiary referral hospitals in low- and middle-income countries due to instrument availability, reagent costs, maintenance requirements, and supply-chain constraints [24]. In such settings, conventional end-point PCR, although less sensitive than qPCR and unable to provide viral load quantification, represents a pragmatic, hospital-based molecular tool that can be integrated into existing laboratory workflows to confirm or refute systemic viremia in serology-reactive inpatients. This approach aligns with antimicrobial stewardship goals by providing an accessible confirmatory test before exposing children to potentially toxic anti-CMV therapy [25].

Local clinical observations at Wahidin Sudirohusodo Hospital indicate 220 CMV cases were recorded in 2023 based on serology, yet the actual incidence of molecularly confirmed active infection remained unquantified. Without evidence-based molecular data and characterization of genuinely viremic patients, the hospital's clinical practice guidelines rely on serologically-based algorithms prone to overdiagnosis and overtreatment. This study was designed to provide molecular identification of CMV DNA in a cohort of pediatric inpatients, thereby establishing a scientific foundation for improved diagnostic algorithms and more rational, efficacious, and safe antimicrobial stewardship at this institution.

## Methods

**Study design:** this was a hospital-based descriptive cross-sectional study conducted among pediatric inpatients admitted to the Pediatric Ward of Wahidin Sudirohusodo Hospital, a tertiary referral and teaching hospital in Makassar, Indonesia, between December 2024 and March 2025.

**Study setting:** this study was conducted from December 2024 to March 2025. Participant recruitment and blood specimen collection were carried out in the Pediatric Ward of Wahidin Sudirohusodo Hospital, Makassar, Indonesia, a tertiary-level teaching and referral hospital. Molecular identification via PCR was performed at the Clinical Microbiology Laboratory, Faculty of Medicine, Hasanuddin University. DNA sequencing was completed at 1st BASE Laboratories Sdn. Bhd., Kuala Lumpur, Malaysia.

**Participants:** the study population comprised all hospitalized children admitted to the pediatric ward during the study period who were clinically suspected of CMV infection by the treating pediatrician and had at least one reactive CMV serological result (IgM and/or IgG). CMV serology in this hospital is typically requested as part of the diagnostic work-up for children presenting with unexplained hepatitis or cholestasis, severe pneumonia, neurological manifestations, sepsis-like illness, or failure to thrive. All eligible patients were included using consecutive (total) sampling, and no randomization procedures were applied during the study period, resulting in a saturated sample of 50 participants.

**Research procedure:** after ethical approval and institutional authorization, approximately 1 mL of peripheral blood was collected by venipuncture into sterile EDTA-anticoagulated tubes using standard aseptic technique, transported on ice, and stored at -20°C until analysis. Only EDTA-anticoagulated whole-blood samples were collected and used for CMV DNA detection in this study due to logistical and resource constraints within the hospital laboratory. Urine and saliva specimens, which are commonly used for diagnosing congenital and early-life CMV infection, particularly in infants, were not included in the protocol and were not routinely obtainable in this setting, a limitation that may have reduced diagnostic sensitivity. DNA extraction was performed using a column-based GSB buffer lysis system. CMV DNA detection was performed using conventional end-point PCR targeting the HindIII-X fragment (406 bp). The following primers (Integrated DNA Technologies, Coralville, Iowa, USA) were used: forward 5'-GGATCCGCATGGCATTCACGTATGT-3' and reverse 5'-GAATTCAGTGGATAACCTGCGGCGA-3'.

Each 25 μ L reaction contained GoTaq Master Mix (12.5 μ L), forward and reverse primers (0.5 μ L each from 10 μ M stocks), template DNA (5 μ L), and nuclease-free water (6.5 μ L). Amplification was performed for 35 cycles (94°C for 1-minute denaturation, 55°C for 2 minutes annealing, and 72°C for 3-minute extension). Amplicons were visualized on 2% agarose gels stained with ethidium bromide by electrophoresis at 100 V for 60 minutes; bands at 406 bp matching the positive control were interpreted as positive. CMV DNA detection was performed using conventional end-point PCR targeting the HindIII-X fragment (406 bp), with visualization by agarose gel electrophoresis. This assay was qualitative and did not provide viral load quantification, and its analytical limit of detection in copies/mL was not experimentally determined in this study. Consequently, negative PCR results were interpreted as indicating CMV DNA levels below the analytic detection threshold of this in-house assay rather than definitive absence of systemic viremia. Quantitative real-time PCR (qPCR), which offers higher analytic sensitivity and permits viral load monitoring, was not available for routine clinical use at the study site during the study period, reflecting diagnostic constraints common in resource-limited tertiary hospitals. PCR-positive samples underwent QIAquick purification and NanoDrop quantification prior to Sanger sequencing (BigDye Terminator v3.1) at 1st BASE Laboratories Sdn. Bhd., Kuala Lumpur, Malaysia. Sequence quality was assessed using FinchTV/Chromas, homology was evaluated using BLASTN (NCBI), and phylogenetic analysis was performed in MEGA using maximum likelihood with 1,000 bootstrap replications.

**Data source:** demographic and clinical data were extracted from medical records. Secondary data captured included age, sex, clinical manifestations, and serological status. Descriptive statistical analysis employed frequency distribution across all variables. Results were stratified by sex, age group, serology profile, and clinical manifestation.

**Study size:** the sample size was estimated using the Harry King nomogram, based on 220 pediatric CMV cases recorded by serology in 2023 at our institution and assuming a 10% margin of error, which yielded a target of 49 subjects (rounded to 50). During the study period, we were able to enroll 50 consecutive eligible children.

**Inclusion and exclusion criteria:** hospitalized children aged 0-17 years of either sex were eligible if they fulfilled all of the following criteria: (i) were admitted to the Pediatric Ward of Wahidin Sudirohusodo Hospital between December 2024 and March 2025; (ii) had been clinically diagnosed with CMV infection by the responsible pediatrician; and (iii) had at least one reactive anti-CMV serological result (IgM and/or IgG). Written informed consent was obtained from parents or legal guardians before enrolment. Children were included irrespective of whether they were IgM/IgG dual positive or IgG only positive, reflecting real-world serology-based diagnostic practice in this high seroprevalence setting. Children with isolated IgG reactivity were not enrolled as evidence of active CMV infection per se, but because they presented with severe or unexplained clinical syndromes in which CMV was considered in the differential diagnosis (e.g., hepatitis or cholestasis, severe pneumonia, neurological manifestations, sepsis-like illness, or failure to thrive) in this tertiary referral setting. In this study, IgG positivity was not interpreted as evidence of active CMV infection.

Exclusion criteria were insufficient blood volume for PCR analysis, visible contamination, hemolyzed or clotted blood, loss or damage of specimen identification labels, or broken/cracked collection tubes.

**Statistical methods:** data were entered into Microsoft Excel and analyzed using descriptive and exploratory inferential statistics. The primary analysis estimated the proportion of serology-reactive hospitalized children with PCR-confirmed CMV DNAemia and described this proportion across sex, age categories, serological patterns, and clinical manifestations. Categorical variables were summarized as counts and percentages and presented in frequency distribution tables. In addition, exploratory Fisher´s exact tests were performed to assess associations between CMV IgM serostatus (IgM-positive vs. IgM-negative) and PCR-confirmed CMV DNA detection. Odds ratios (ORs) and exact p-values were calculated for 2x2 contingency tables where cell counts permitted, with statistical significance defined as p <0.05. Given the modest sample size and the limited number of PCR-positive events, these inferential analyses were regarded as hypothesis-generating rather than definitive.

**Ethical considerations:** the Ethics Team of Hasanuddin University has examined and approved this research. This research was conducted in accordance with all applicable requirements. Ethical approval was obtained from the Institutional Review Board of the Faculty of Medicine, Hasanuddin University (approval number: 448/UN4.6.4.5.31/PP36/2024, issued on June 14, 2024). All procedures followed the principles of the 1964 Declaration of Helsinki and its later amendments, Good Clinical Practice guidelines, and the International Conference on Harmonization standards.

## Results

**Demographic characteristics and PCR detection by sex:** of 50 hospitalized children, 28 (56%) were male, and 22 (44%) were female. Overall, 9 (18%) were positive for CMV DNA, and 41 (82%) were negative. PCR positivity was higher in males: 6 of 28 (21.4%) versus 3 of 22 females (13.6%), indicating a male predominance in active viremia detection (Table 1).

**Table 1:** cytomegalovirus DNA detection by blood PCR among hospitalized children, recruited from the pediatric ward of Wahidin Sudirohusodo Hospital, Makassar, Indonesia, from December 2024 to March 2025 (N=50)

Characteristic	Category	n (%)	PCR positive n (%)	PCR negative n (%)
Sex	Male	28 (56.0)	6 (21.4)	22 (78.6)
	Female	22 (44.0)	3 (13.6)	19 (86.4)
Age	<1 year	29 (58.0)	7 (24.1)	22 (75.9)
	1-4 years	10 (20.0)	1 (10.0)	9 (90.0)
	5-6 years	1 (2.0)	0 (0.0)	1 (100.0)
	7-17 years	10 (20.0)	1 (10.0)	9 (90.0)
Serology	IgM+/IgG+	10 (20.0)	5 (50.0)	5 (50.0)
	IgM-/IgG+	39 (78.0)	4 (10.3)	35 (89.7)
	IgM+/IgG-	1 (2.0)	0 (0.0)	1 (100.0)
Clinical manifestation	Hepatic	32 (64.0)	6 (18.8)	26 (81.3)
	Respiratory	9 (18.0)	1 (11.1)	8 (88.9)
	Neurological	8 (16.0)	2 (25.0)	6 (75.0)
	Cardiac	1 (2.0)	0 (0.0)	1 (100.0)

PCR: polymerase chain reaction

**Polymerase chain reaction detection by age group:** the cohort was distributed as follows: infants <1 year (n=29, 58%), toddlers 1-4 years (n=10, 20%), preschool 5-6 years (n=1, 2%), and school-age/adolescent 7-17 years (n=10, 20%). The highest CMV DNA detection rate occurred in infants: 7 of 29 (24.1%). Toddlers and school-age children showed equal detection rates of 10% (1 of 10 in each group). The single preschool-aged child was PCR-negative (Table 1).

**Polymerase chain reaction detection by serological status:** stratification by antibody profile revealed: IgM/IgG dual-positive (n=10): 5 positives (50%); IgM-negative/IgG-positive (n=39): 4 positives (10.3%); IgM-positive/IgG-negative (n=1): 0 positives (0%). The substantial difference in detection rates between dual-positive and IgG-only patterns demonstrates that concurrent IgM and IgG positivity shows the strongest association with active viremia, while isolated IgG reactivity indicates historical or latent infection with minimal active viral replication (Table 1).

Exploratory association between IgM positivity and PCR positivity. In an exploratory Fisher´s exact test, IgM positivity (IgM-positive vs. IgM-negative) was associated with higher PCR positivity: 5 of 11 IgM-positive children were PCR-positive, compared with 4 of 39 IgM-negative children. The odds ratio was 7.29 with a two-sided Fisher´s exact p-value of 0.017. Given the small sample size and low number of PCR-positive events, this result should be regarded as hypothesis-generating rather than definitive. Despite this lower positive predictive value, children with isolated IgG positivity were included in molecular testing because they were hospitalized with severe or unexplained clinical syndromes in which CMV remained a plausible etiologic consideration in this tertiary referral setting.

**Polymerase chain reaction detection by clinical manifestation:** hepatic manifestations were most common (n=32), with CMV DNA detected in 6 (18.8%). Respiratory involvement (n=9) showed DNA detection in 1 (11.1%). Neurological manifestations (n=8) had the highest proportional detection at 2 (25.0%). Cardiac manifestation (n=1) was entirely negative. These findings illustrate that clinical presentation does not reliably predict molecular viremia, with the majority of symptomatic cases (82%) showing no detectable blood CMV DNA. Detailed clinical manifestations are presented in Table 1.

**Figure 1:**
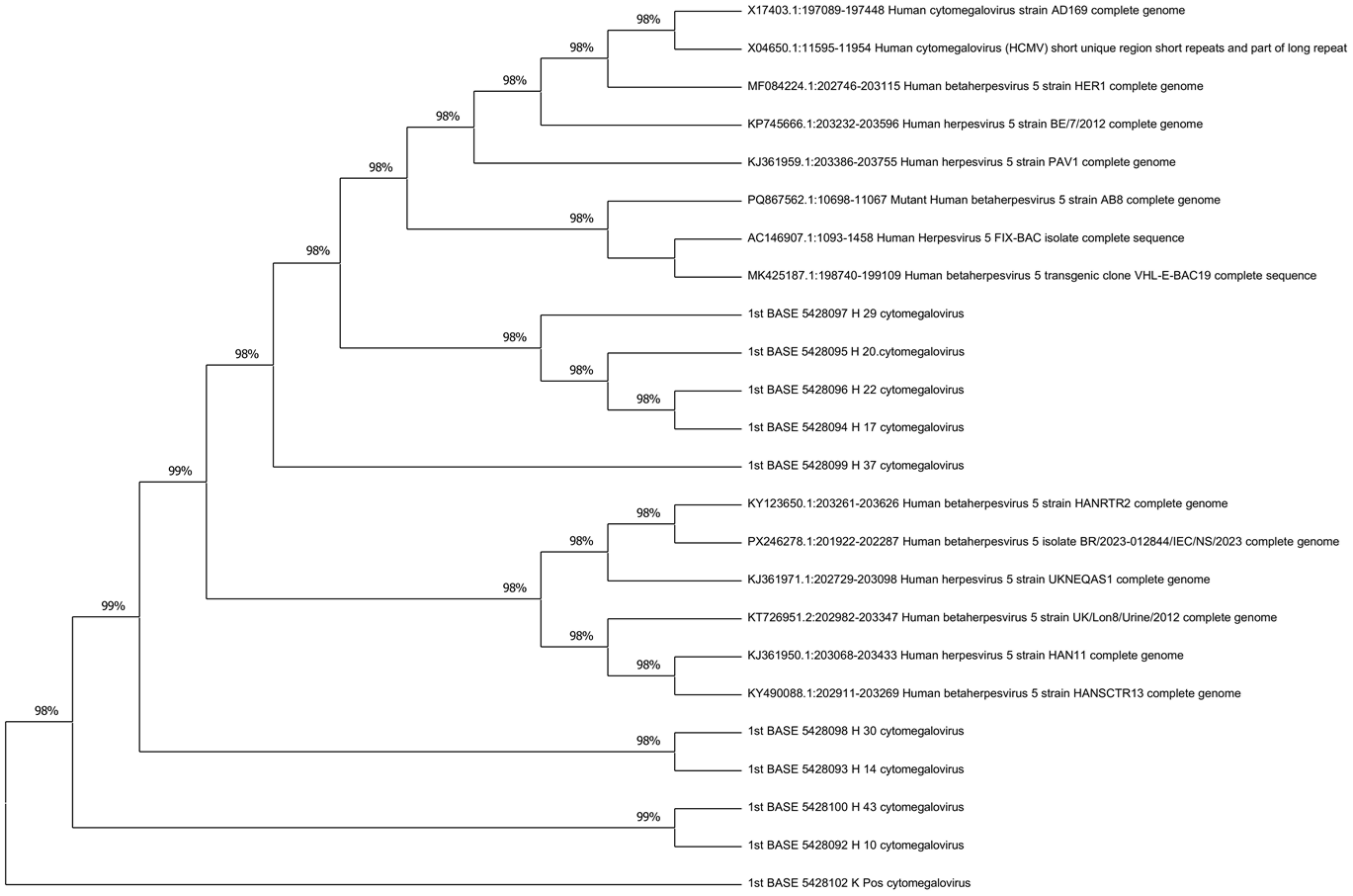
phylogenetic tree of CMV sequences from hospitalized children, recruited from Wahidin Sudirohusodo Hospital, Makassar, Indonesia, from December 2024 to March 2025 (N=9)

**Phylogenetic classification:** all 9 PCR-positive samples, along with the positive control, clustered monophyletically within human betaherpesvirus 5 species, confirming HCMV identity. Phylogenetic topology revealed two major clades with high bootstrap support (98-99%): clade I (samples H17, H20, H22, H29, H37) and clade II (samples H10, H14, H30, H43). This separation indicates the presence of more than one closely related sequence cluster within the study population, reflecting limited genetic variability among the CMV strains sampled in this hospital cohort and providing a descriptive snapshot consistent with previously reported global HCMV heterogeneity (Figure 1).

## Discussion

This study evaluated the presence of active CMV infection via molecular detection in 50 hospitalized pediatric patients previously identified through serological screening. The principal finding that only 18% of serologically positive cases showed PCR-confirmed viremia has substantial implications for pediatric diagnostic practice and antiviral stewardship.

**Sex-based differences in viremia detection**: the higher detection rate observed in male patients (21.4% vs. 13.6% in females) may reflect sex-based differences in immune control of CMV infection. These differences have been attributed to two main mechanisms. First, females possess two X chromosomes enriched with immune-related genes, some of which escape X-chromosome inactivation (XCI), potentially enhancing antiviral responses compared with males. Second, CD8^+^cytotoxic T-cell responses, which are critical for controlling CMV viremia, have been reported to develop more slowly in males, potentially prolonging detectable viremia and increasing the likelihood of PCR positivity. Collectively, these immunological differences, which have been described in pediatric infectious diseases, suggest the need for further investigation into sex-based differences [26,27]. However, individual immune status variables such as HIV infection, primary immunodeficiency, or exposure to immunosuppressive therapies were not systematically documented in this cohort, so these mechanistic explanations should be regarded as hypothesis-generating rather than causally demonstrated by our data.

**Age-related epidemiology**: the highest prevalence of active infection in infants <1 year (24.1%) is consistent with global epidemiological patterns showing higher CMV burden in developing countries, where birth prevalence ranges from 0.6% to 6.1% compared to 0.2-2.0% in developed countries. Two plausible mechanisms may contribute to this higher incidence: late-manifesting congenital infection and early postnatal transmission via breast milk from seropositive mothers. Breast milk from CMV-positive mothers represents a major source of infant CMV acquisition, with transmission rates exceeding 60% in breastfed infants during the first year of life, with sustained viral replication and blood detectability until immune maturation [28]. Immunologically, infant immune immaturity impairs CD8^+^ T cell and natural killer cell function, reducing viral clearance capacity and prolonging viremia, thus facilitating molecular detection [29]. By school age, improved adaptive immunity suppresses reactivation to latency (10% detection), explaining the age-dependent trend [29].

**Serological-molecular discordance**: marked disparity between serology and PCR-50% concordance (dual-positive) versus 10.3% (IgG-only)-reflects fundamental diagnostic limitations. CMV-IgG values vary 185-fold across assays with 32.9% sensitivity for primary infection; EBV-CMV IgM dual positivity occurs in 25% of primary EBV cases yet represents true CMV coinfection in only 2.5%-5%, indicating 95-97.5% false-positives from cross-reactivity [30]. Relying on serology to initiate antiviral therapy (ganciclovir/valganciclovir) risks unnecessary drug exposure with substantial morbidity (neutropenia 25-60%, thrombocytopenia, nephrotoxicity) [31]. Serial IgG retesting at 3-5 weeks provides minimal utility and may mislead; serological kinetics-based diagnosis is fundamentally unreliable [31]. PCR confirmation is mandatory before therapy to distinguish active viremia from latent infection or false-positive serology [30,31].

Beyond biological explanations, the high proportion of PCR-negative cases is also likely influenced by the analytical performance of our conventional endpoint PCR assay. Conventional PCR is a qualitative method that reports the presence or absence of amplification of a target sequence, and a negative result reflects failure to detect viral DNA above the assay´s limit of detection rather than definitive absence of virus [32]. Comparative evaluations of molecular assays have shown that the analytical sensitivity of RT PCR systems can differ within an 8-fold range (approximately 100-800 copies/mL), with some optimized rapid or real-time formats achieving lower limits of detection than conventional assays [33]. In our setting, the analytical limit of detection of the in-house conventional PCR used for CMV was not formally quantified, so negative results should be interpreted as “no CMV DNA detected above this assay´s detection threshold”, particularly in children with low-level or compartmentalized infection.

In an exploratory Fisher´s exact test, IgM positivity (IgM-positive vs. IgM-negative) was associated with higher PCR positivity: 5 of 11 IgM-positive children were PCR-positive, compared with 4 of 39 IgM-negative children. The odds ratio was 7.29 with a two-sided Fisher´s exact p-value of 0.017. Given the small sample size and low number of PCR-positive events, this result should be regarded as hypothesis-generating rather than definitive. This finding is biologically plausible, as IgM positivity often reflects more recent or ongoing immune stimulation and has been associated with a higher likelihood of concurrent viral DNA detection or shedding in pediatric CMV cohorts [34]. At the same time, CMV IgM is known to be imperfect and prone to persistence and cross-reactivity, including with Epstein-Barr virus, which limits its specificity as a standalone marker of primary infection [35]. Given the small number of IgM-positive and PCR-positive cases and the cross-sectional, descriptive design, this association should not be overinterpreted; rather, it provides preliminary support for prioritizing IgM-reactive children, particularly those with combined reactive IgM/IgG profiles, for molecular confirmation when resources are limited, while recognizing the inherent limitations of IgM-based screening. Taken together, these findings are compatible with at least two non-mutually exclusive explanations for the high proportion of PCR-negative yet clinically symptomatic cases: compartmentalized CMV replication within specific organs that is not accompanied by substantial systemic DNAemia, and the limited analytical sensitivity of our qualitative conventional PCR assay for detecting low-level viremia. The relative contribution of these biological and methodological factors cannot be determined from this cross-sectional dataset [34,35].

**Serological-molecular temporal disconnect**: blood collection (0-2 days´ post admission) versus symptom onset (weeks-months prior) complicates interpretation; DNAemia may decline to undetectable despite robust serological responses coinciding with minimal blood CMV DNA. CMV IgM peaks then declines rapidly, extending seropositivity after viral clearance. IgG avidity clarifies infection timing: low avidity (<40-50%) indicates recent infection (3-4 months), while high avidity (>60%) indicates remote infection (>3-4 months). Avidity transitions from low to high within 2-4 months, enabling diagnostic precision and biologically consistent infection dating regardless of specimen delays, superior to PCR for temporal resolution [35,36].

**Clinical manifestation-viremia mismatch**: importantly, clinical syndromes in this study were extracted from medical records and were not adjudicated using standardized criteria for CMV end-organ disease; therefore, organ-specific interpretations should be considered hypothesis-generating. The 82% PCR negativity most likely reflects a combination of viral compartmentalization with localized organ replication and methodological limitations of our blood-based conventional PCR assay, resulting in minimal detectable systemic viremia in many clinically symptomatic children. In children with hepatic manifestations, tissue-invasive CMV disease cannot be confirmed in our cohort because no liver biopsy or tissue-based CMV testing was performed. Nevertheless, prior literature suggests that CMV may be detected in liver tissue in a subset of pediatric cholestasis/hepatitis cases and that blood CMV PCR can be negative despite organ involvement. Therefore, blood PCR negativity in our study should not be interpreted as excluding compartmentalized hepatic infection, particularly when using conventional end-point PCR [37]. In children with respiratory manifestations, our data cannot establish CMV pneumonitis because respiratory specimens (e.g., bronchoalveolar lavage) were not collected, and standardized definitions of tissue-invasive CMV lung disease were not applied. Blood PCR may underestimate respiratory CMV replication, and detection of CMV in airway specimens, together with compatible clinical and radiologic findings, has been used in other settings to support probable CMV pneumonia. However, co-infections are common in hospitalized children and may account for respiratory symptoms in many cases, which warrants cautious interpretation [37,38]. Neurological disease shows the highest blood PCR positivity (25%), yet 75% of cases remain blood PCR-negative, indicating persistent CNS damage despite blood viral clearance, characteristic of chronic or previous infection. The cardiac case's PCR-negativity, combined with uncommon primary CMV myocarditis in immunocompetent children, raises the possibility that some cardiac manifestations may be attributable to other pathogens such as enterovirus or adenovirus, although this could not be confirmed [37,38]. However, because tissue-based diagnostics such as liver biopsy, bronchoalveolar lavage, or cerebrospinal fluid PCR were not systematically performed, these organ-specific interpretations remain speculative. In particular, we cannot distinguish with certainty between CMV-related inflammation and alternative or co-existing etiologies in children with hepatic, respiratory, or neurological involvement [37,38].

**Implications for antimicrobial stewardship**: in this tertiary hospital, 220 pediatric CMV cases were recorded in 2023 based solely on serology, raising concern for potential overdiagnosis and overtreatment. Given the toxicity profile of ganciclovir and valganciclovir, including neutropenia, thrombocytopenia, and nephrotoxicity, initiating antiviral therapy without molecular evidence of systemic viremia may expose children to avoidable harm and contribute to healthcare resource strain. Our findings support embedding molecular confirmation (even using conventional PCR when qPCR is unavailable) into diagnostic algorithms, particularly for IgM-positive and clinically severe cases. Such an approach aligns with emerging antiviral stewardship frameworks, seeking to target therapy to genuinely infected patients while minimizing unnecessary drug exposure [39]. Phylogenetic insights: within the limitations of our small sample (n=9), the phylogenetic tree showed two sequence clusters with high bootstrap support (98-99%), suggesting that at least two closely related CMV variants were circulating in this hospital during the study period. This pattern is consistent with the well-described genomic heterogeneity of HCMV, where variable regions often cluster into phylogenetic groups. In our study, the phylogenetic analysis was descriptive in nature and was not designed to assess strain-specific virulence or clinical correlates. While envelope glycoprotein diversity may influence immunogenicity and cross-strain neutralization, the present dataset is too limited to draw conclusions regarding strain-specific pathogenicity, and larger sequencing studies will be required to clarify how HCMV genetic variants contribute to clinical outcomes [40].

**Limitations:** this study has several important limitations. First, its single-center, hospital-based design in a tertiary referral setting limits generalizability beyond similar high-seroprevalence, referral-hospital populations and warrants multicenter prospective validation. Second, molecular testing relied exclusively on ethylenediaminetetraacetic acid (EDTA) whole-blood specimens; this approach is less sensitive than combined saliva or urine testing, particularly for congenital and early postnatal CMV infection in infants, and may have underestimated the true burden of active infection. Third, conventional end-point PCR was used instead of quantitative real-time PCR (qPCR); the absence of viral load quantification and the inability to define an exact analytic limit of detection in copies/mL, together with the generally lower analytical sensitivity of conventional assays, limit interpretation of negative results, especially in cases of low-level or compartmentalized infection. Fourth, IgG avidity testing and serial PCR measurements were not performed, precluding temporal characterization of the infection phase and viral kinetics. Fifth, the descriptive, cross-sectional design limited adjustment for potential confounders, and inferential analysis was restricted to exploratory associations using Fisher´s exact test. Finally, detailed host-related factors-including immunological status, HIV infection, immunosuppressive therapy, and maternal serology-were not systematically available, limiting mechanistic interpretation of why detectable viremia occurred in only a subset of serologically positive children.

## Conclusion

This study demonstrates that 18% of serologically positive pediatric CMV cases show molecular confirmation of active infection via PCR, with striking male predominance, highest prevalence in infants, and strongest correlation with dual IgM/IgG serology. The 82% discordance between serology and molecular findings underscores the inadequacy of antibody-based diagnosis alone. Molecular confirmation via PCR should be considered as a confirmatory standard prior to antiviral therapy initiation, particularly in IgM-positive cases, to prevent unnecessary treatment toxicity and optimize clinical outcomes. Phylogenetic analysis confirmed HCMV identity and demonstrated limited genetic variability within at least two closely related sequence clusters, suggesting the coexistence of multiple closely related circulating variants in this setting.

### What is known about this topic

- Serological diagnosis of CMV infection exhibits limited specificity in pediatric populations, particularly in high-seroprevalence settings;
- Active CMV infection is associated with substantial morbidity and mortality in vulnerable pediatric groups, including neonates and immunocompromised hosts;
- Antiviral therapies for CMV carry significant hematologic and renal toxicity, requiring careful assessment of the benefit-risk balance before initiation.

### What this study adds

- Quantification of active CMV viremia among serologically positive hospitalized children reveals 82% may lack detectable blood virus, suggesting overdiagnosis via serology alone;
- Male children and infants <1 year represent the highest-risk groups for molecular-confirmed active infection;
- Concurrent IgM/IgG positivity shows the strongest association (50%) with active viremia, while IgG alone carries a low positive predictive value (10.3%), providing serological stratification guidance for molecular testing priority.
